# *Toxoplasma gondii*, endothelial cells and schizophrenia: is it just a barrier matter?

**DOI:** 10.3389/fcimb.2025.1468936

**Published:** 2025-04-10

**Authors:** Victoria Cruz Cavalari, Luiz Fernando Cardoso Garcia, Raffael Massuda, Letusa Albrecht

**Affiliations:** ^1^ Laboratório de Pesquisa em Apicomplexa – Instituto Carlos Chagas, Fundação Oswaldo Cruz, Curitiba, Paraná, Brazil; ^2^ Departamento de Medicina Forense e Psiquiatria da Universidade Federal do Paraná, Curitiba, Paraná, Brazil

**Keywords:** toxoplasmosis, schizophrenia, endothelial cell, neuroinflammation, *Toxoplasma gondii*

## Abstract

*Toxoplasma gondii* is an obligatory intracellular parasite responsible for causing toxoplasmosis. It is estimated that approximately one-third of the world’s population has positive serology for toxoplasmosis. Acute *T. gondii* infection often results in subtle symptoms because of its nonspecific nature. Owing to immune pressure, parasites tend to encyst and persist in different tissues and organs, such as the brain, chronicling the infection. While most chronically infected individuals do not develop significant symptoms, the parasite can affect the central nervous system (CNS), leading to symptoms that range from dizziness to behavioral changes. To reach the CNS, parasites must overcome the blood–brain barrier, which is composed primarily of endothelial cells. While these cells are typically efficient at separating blood elements from the CNS, in *T. gondii* infection, they not only permit parasitic colonization of the CNS but also contribute to an inflammatory profile that may exacerbate previously established conditions at both the local CNS and systemic levels. An increasing body of research has demonstrated a potential link between the CNS, infection by *T. gondii* and the cellular or humoral response to infection, with the worsening of psychiatric conditions, such as schizophrenia. Therefore, continually advancing research aimed at understanding and mitigating the relationship between parasitic infection and schizophrenia is imperative.

## Introduction

*Toxoplasma gondii* (*T. gondii*) is a parasite belonging to the phylum Apicomplexa. It is the etiological agent of toxoplasmosis, a disease that holds considerable public health significance, largely because of its morbidity. Approximately 30% of the global human population carries *T. gondii* (Safarpour et al., 2020). In healthy individuals, toxoplasmosis often remains asymptomatic. However, immunocompromised individuals can experience various manifestations ranging from headaches and elevated liver enzymes to lymph node enlargement, fever, pneumonia, and even central nervous system (CNS) involvement (Bossi et al., 1998; Carme et al., 2002).

During *T. gondii* infection, the parasite has the capacity to breach the endothelial barrier, allowing it to infiltrate various organs or tissues, including the brain (Lachenmaier et al., 2011; Konradt et al., 2016; Baba et al., 2017). Neurotoxoplasmosis is characterized by the presence of *T. gondii* cysts in the brains of infected individuals (Mesquita et al., 2010; Alvarado-Esquivel et al., 2015). Several studies have evaluated neurological changes caused by *T. gondii* infection, and one of the most studied relationships is between *T. gondii* infection and schizophrenia (Alvarado-Esquivel et al., 2011; Dickerson et al., 2014). Although the relationship between *T. gondii* infection and neurological disorders has been studied for decades (Navia et al., 1986; Khalid et al., 2023), the precise relationship between *T. gondii* infection and the development of schizophrenia remains uncertain.

Given the significance attributed to toxoplasmosis and its potential correlation with the onset or exacerbation of psychiatric disorders, we have undertaken this literature review to ascertain the current state of research pertaining to toxoplasmosis, the CNS endothelium and its influence on the progression of schizophrenia.

## Endothelium

Endothelial cells derived from the embryonic mesoderm are vital for body homeostasis (Zovein et al., 2008). Although the endothelium shares a common origin, it exhibits significant heterogeneity throughout the human body, as will be briefly discussed in this section.

The endothelium can be classified as continuous when its cells are joined by tight junctions, forming a protective barrier, as seen in blood vessels, the heart, lungs, skin, and the central nervous system (CNS). The fenestrated endothelium, with transcellular pores, allows solute exchange in organs like the kidneys and liver. In contrast, the fenestrated endothelium, featuring larger gaps or spaces, enables the passage of large molecules and is found in the sinusoids of the liver, bone marrow, and spleen (Margreet De Leeuw et al., 1990; Price et al., 2020; Su et al., 2021; Zuo et al., 2021). Functionally, it regulates vascular permeability and produces vasoactive substances such as nitric oxide and prostacyclin, influencing blood flow and pressure (Iwabayashi et al., 2012; Mitchell et al., 2021). Dysfunction contributes to cardiovascular diseases such as atherosclerosis, thrombosis, and hypertension by altering permeability, promoting inflammation, and impairing vasodilation (Siragusa et al., 2021; Jiang et al., 2022).

Endothelial heterogeneity can also be found within the brain. For instance, in the cerebral ventricles, there is a structure called the choroid plexus, which is responsible for secreting cerebrospinal fluid (CSF). This organ consists of epithelial cells and fenestrated endothelial cells, characterized by the presence of diaphragms (Schmidley and Wissig, 1986). In addition to its role in forming the blood-CSF barrier, the choroid plexus has recently gained attention as a potential site for the initial infection of *T. gondii* in the brain (Figueiredo et al., 2022).

Cerebral endothelial cells are part of the blood–brain barrier (BBB) (Poller et al., 2008; Urich et al., 2012), which consists of multiple associated components, such as pericytes and perivascular astrocytes. Together with neurons, oligodendrocytes and microglia, these components form the neurovascular unit (NVU), which maintains a stable neural environment, regulates cerebral blood flow and protects the brain from toxins (Bobot et al., 2020; Nagata et al., 2021).

Brain microvascular endothelium cells (BMECs) are connected by tight junctions, they lack fenestrations, show minimal pinocytic activity, and have high electrical resistance (TEER), selective permeability (Omran et al., 2020; Sun et al., 2022). BMECs regulate nutrient exchange, modulate immune responses, and influence neuroinflammation via cell adhesion molecules (Mugisho et al., 2020). Single-cell RNA sequencing highlights endothelial heterogeneity (Liu et al., 2021). Under some conditions, immortalized human cerebral microvascular endothelial cells (hCMEC/D3) upregulate MHC class II, reflecting immune shifts (Wheway et al., 2013; Lopes Pinheiro et al., 2016).

Different molecules can modulate gene expression in endothelial cells, ranging from proinflammatory molecules, such as prostaglandins (Wilhelms et al., 2014) to extracellular histones (Pérez-Cremades et al., 2017). Some established molecules for endothelial activation are interleukin 1 beta, tumor necrosis factor alpha (TNF-α) and lipopolysaccharide (Makó et al., 2010; Jiang et al., 2015; Jiang et al., 2016). Molecules such as cytokines (Bautista et al., 2005), soluble proteins (Sasongko et al., 2014), vascular growth factors (Waheed et al., 2016), elements involved in blood clotting (Jiang et al., 2016) and oxidative stress (Couillard et al., 2005) are commonly identified as inflammatory markers (Drenjancevic et al., 2018).

Endothelial changes can be caused by various infectious agents (De Assis et al., 2000; Knight et al., 2005; Debrah et al., 2007; Liu et al., 2010; Calvert et al., 2015). During dengue virus infection, the endothelium produces large concentrations of IL-8 and initiates the apoptosis process (Avirutnan et al., 1998). In human immunodeficiency virus infection, high serum levels of von Willebrand factor and tissue plasminogen activator resulting from endothelial activation are observed (De Larrañaga et al., 2003). Endothelial cells infected by *Chlamydia pneumoniae* are activated by increased expression of adhesion molecules such as intercellular adhesion molecule 1 (ICAM-1), vascular cell adhesion protein 1 (VCAM-1), E-selectin and mitogen-activated protein kinase (Krüll et al., 1999). Brain endothelial cells infected by *Streptococcus pneumoniae* express high levels of IL-8 and the chemokines CXCL-1 and CXCL-2 in response to bacterial neuroaminidases (Banerjee et al., 2010).

It is important to emphasize that, despite their significant role, endothelial cells do not perform their functions alone. For example, the loss or degeneration of pericytes (cells located around blood vessels) disrupts cerebral blood flow, leading to an imbalance in neural function (Nikolakopoulou et al., 2019; Hartmann et al., 2021). Additionally, Menard et al. demonstrated that pericyte-endothelial communication plays a crucial role in regulating emotional responses to stress and anxiety, as pericyte ablation resulted in these behaviors (Menard et al., 2024).

As a cascade effect, for the endothelium-pericyte interaction to occur, astrocytic end-feet contribute to the maintenance and regulation of pericyte differentiation, while also modulating the expression of endothelial cell tight junction proteins such as claudin-5 (CLDN5) and ZO-1 (Heithoff et al., 2021a; Heithoff et al., 2021b). In addition, to their well-known role in endothelial cell function, pericytes have also been shown to play a part in the immune response. *In vitro* studies have demonstrated that, in response to inflammatory stimuli, pericytes produce reactive oxygen species, modulate phagocytosis, and regulate the expression of α-smooth muscle actin and ICAM-1 (Pieper et al., 2014).

Thus, the integrity of this neurovascular unit is crucial for proper brain function, and any disruption in this system can lead to diseases, as will be discussed in later sections.

## *Toxoplasma gondii* and the blood-brain barrier

*T. gondii* is a zoonotic protozoan of the phylum Apicomplexa (Dubey, 2008), discovered in 1908 in *Ctenodactylus gundi*, and it is capable of infecting different mammals, including humans (Sabin and Olitsky, 1937; Dubey et al., 1997; Dubey, 2008). The life cycle involves sexual reproduction in feline enterocytes due to their lack of the enzyme D6D, leading to the accumulation of linoleic acid, which is essential for *T. gondii* reproduction (Davidson and Haggan, 1990; Di Genova et al., 2019).

Felids ingest tissue cysts, which release trophozoites in the intestine. These invade epithelial cells, undergo asexual replication, and form gametocytes, leading to oocyst production. Oocysts are subsequently excreted in feces and sporulate into resistant forms that can persist in the environment for more than a year (Webster, 1994; Lélu et al., 2012; Hehl et al., 2015). Intermediate hosts, such as rodents, sheep, and humans, ingest oocysts, where tachyzoites invade cells, differentiate into bradyzoites, and form cysts, restarting the cycle upon ingestion by cats (Dubey et al., 1997; Dubey, 2007; Moraes Lm de et al., 2011).

**Figure 1:**
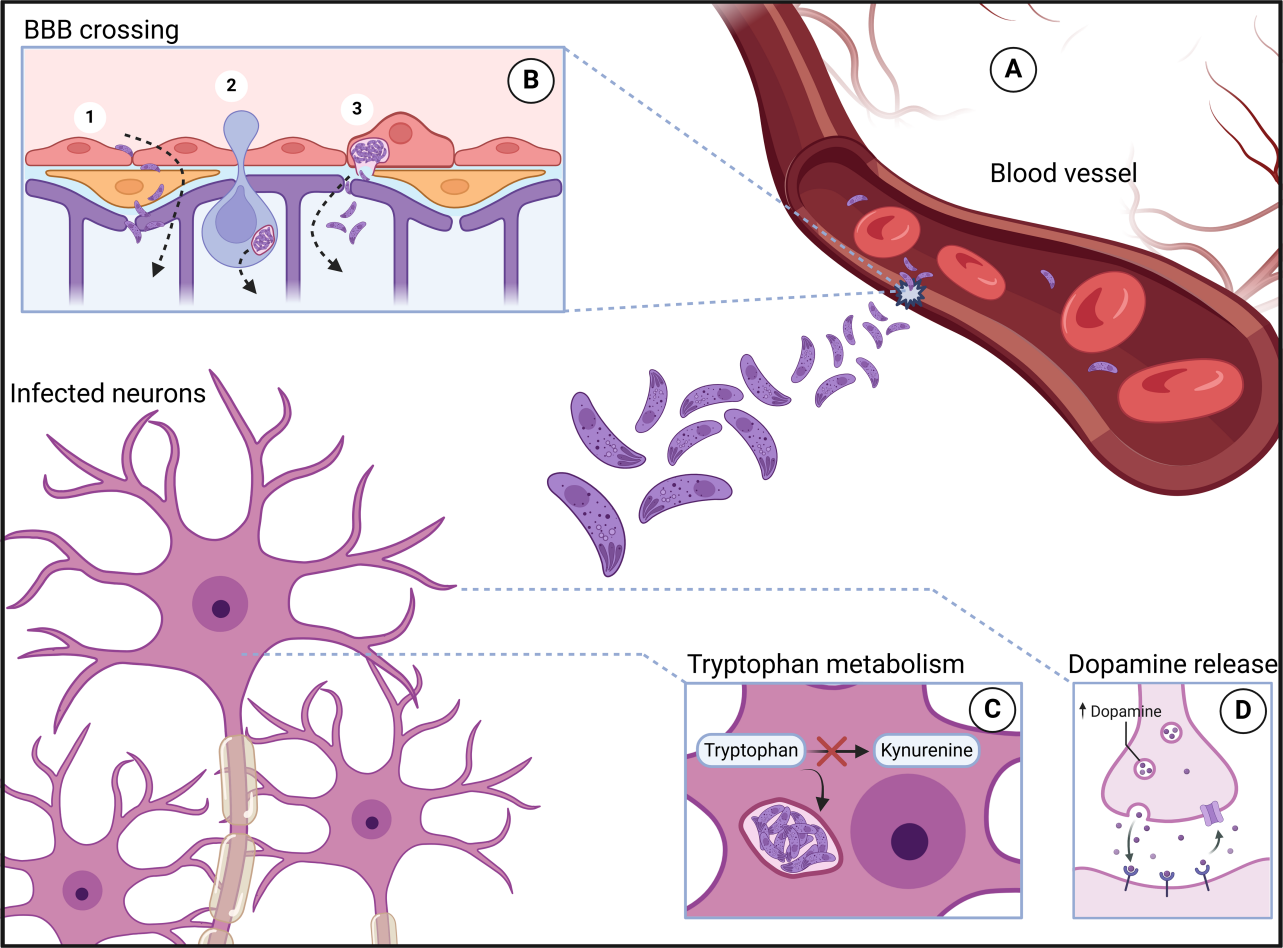
*Toxoplasma gondii* infection. After trophozoites leave the intestine, they enter the bloodstream **(A)**, where they reach various anatomical sites, including areas with significant neurotropism. In these locations, the parasites are able to cross the blood–brain barrier **(B)** through mechanisms such as paracellular passage (1B), the Trojan horse mechanism (2B), and transcellular passage (3B). Once inside neurons, the parasites induce several changes, including rerouting of the tryptophan metabolic pathway toward the parasite, which leads to a reduction in serotonin production **(C)** and an increase in dopamine release **(D)**. Created in BioRender. Albrecht, L. (2024) https://app.biorender.com/citation/673d2af55625e5565695ea24.

Key clinical complications include neurotoxoplasmosis (Carme et al., 2002; Alvarado-Esquivel et al., 2015) and congenital toxoplasmosis (Da Silva et al., 2015; Maldonado et al., 2017). The latter occurs when *T. gondii* crosses the placenta during pregnancy, causing chorioretinitis, intracranial calcification, and hydrocephalus (Maldonado et al., 2017). With up to 80% incidence in endemic areas, diagnosis is challenging owing to nonspecific signs in most cases (Montoya and Remington, 2008; Da Silva et al., 2015). To invade the brain, *T. gondii* must leave the bloodstream and cross the BBB. To overcome this barrier, different mechanisms have been proposed, including those involving paracellular, transcellular and infected immune cells. Different lines of study strongly suggest that *T. gondii* can use all routes (Daneman and Rescigno, 2009; Lachenmaier et al., 2011; Konradt et al., 2016) (**Figure 1**).

The paracellular route involves the passage of a pathogen through the junctions between endothelial cells. Although *T. gondii* lacks a flagellum for mobility, it moves via actin and myosin in a process called gliding motility (Dobrowolski and Sibley, 1996). *T. gondii* is capable of overcoming the intestinal epithelium via the paracellular route (Weight and Carding, 2012), and since this epithelium has similarities with the BBB, invasion of the CNS via this route has become plausible. *T. gondii* can also cross the BBB through the transcellular route in a murine model, invading cells and traversing them without promoting significant damage or inducing lysis. Additionally, the parasite can use endothelial cells as a replicative niche (Konradt et al., 2016; Figueiredo et al., 2022).

Leukocytes infected with *T. gondii* exhibit increased motility and the ability to cross the BBB under static or flowing culture conditions (Lachenmaier et al., 2011; Harker et al., 2013; Ueno et al., 2014). Another study carried out in the pulmonary endothelium revealed a mixture of *T. gondii*-infected leukocytes via the transcellular route. In this work, the authors observed that *T. gondii* leaves the host leukocyte once it is close enough to an endothelial cell that would host the protozoan (Baba et al., 2017). Once the parasite reaches the brain, it can invade any nucleated cell. Interestingly, *in vitro*, the parasite can establish a persistent infection in any of these cells (Fischer et al., 1997). However, *in vivo*, infection and its maintenance, in the form of cysts, can be observed only in neurons. The most intriguing finding is that studies have shown not only that neurons are unable to resolve infection, similar to other neural cells but also that they are the primary target of infection by this parasite (Cabral et al., 2016). In this context, successful elimination of the parasite relies on the synergistic action of IFN-γ and TNF-α produced by immune cells (Scharton-Kersten et al., 1997; Ibrahim et al., 2009). When in contact with the target cell, these inflammatory molecules promote the action of indoleamine-2-3 dioxygenase, iNOS and eNOS (Däubener et al., 2001; Dincel and Atmaca, 2015). Indoleamine-2-3 dioxygenase acts by catabolizing cellular tryptophan and deprives *T. gondii* of this essential amino acid, which results in elimination of the parasite (Däubener et al., 2001). eNOS activity in endothelial cells promotes the formation of nitrites, anions, superoxides and nitric oxide. These molecules have the potential to inhibit the development of *T. gondii* in macrophages and fibroblasts when they act together with other harmful agents to the parasite (Woodman et al., 1991). iNOS-knockout (iNOS-/-) mice infected with *T. gondii* presented increased tissue parasitism and an inflammatory reaction in the CNS (Silva et al., 2009). These findings suggest that reactive oxygen species play a neuroprotective role in *T. gondii* infections. On the other hand, there is evidence that reactive oxygen species triggered by different factors can promote weakening of the BBB (Bao and Shi, 2010) and intensify encephalitis in infected mice (Dincel and Atmaca, 2015). Therefore, reactive oxygen species seem to have an ambiguous effect on neurotoxoplasmosis.

IFN-γ influences various cells, leading to the upregulation of iNOS, a pivotal factor in the clearance of intracellular tachyzoites, particularly within the CNS, which is achieved by inhibiting parasitic mitochondrial function (Scharton-Kersten et al., 1997; Ibrahim et al., 2009). The regulatory function of iNOS is facilitated by the production of anti-inflammatory interleukins, such as IL-10, by cells such as CD4^+^ Foxp3^+^ T (Treg) cells, thereby fostering host homeostasis (Oldenhove et al., 2009). Additionally, IFN-γ secreted by brain-resident cells enhances the expression of molecules involved in T-cell immunity, such as chemokines (CXCL9, CXCL10 and CXCL11), MHC I and MHC II, ICOSL costimulatory molecules and cytokines, including IL-12, IL5- and IL-18 (Suzuki et al., 2023).

MCP1, also known as CCL-2, is a monocyte chemoattractant (Deshmane et al., 2009). Its production is associated with inflammation, monocytic infiltrates and apoptotic events (Gerszten et al., 1999; Zhang et al., 2011). In the context of endothelium infection, the expression of MCP1 varies according to the infectious agent. MCP1 is upregulated in hepatitis C virus infection (Liu et al., 2016), and its expression is basal in *Cryptococcus neoformans* infection (Jong et al., 2008). In endothelial cells infected by *T. gondii*, the expression of MCP1 is maintained for up to 24 hours after the onset of infection (Knight et al., 2005). The parasite surface antigen glycoprotein 1 (SAG1) is sufficient to stimulate MCP1 expression in endothelial cells (Brenier-Pinchart et al., 2006). An imbalance in MCP1 synthesis may be associated with neuroinflammatory processes (Sawyer et al., 2014).

ADAMTS-13 is produced by endothelial cells. Its main function is related to coagulation; however, this molecule has a neuroprotective role by regulating von Willebrand factor activity, preventing microvascular thrombosis and reducing oxidative stress (Fujioka et al., 2010; Khan et al., 2012). High levels of ADAMTS-13 were observed in the brains of *T. gondii*-infected animals at 10, 30 and 60 days post infection, and a potential neuroprotective role was postulated (Dincel and Atmaca, 2016). The infection of human BMECs by *T. gondii* is reduced after the use of drugs such as monensin, which interferes with the host cell cycle, DNA synthesis and repair mechanisms (Harun et al., 2020).

Cerebral toxoplasmosis is characterized by the successful infiltration of *T. gondii* into the CNS. Once situated, tissue cysts containing protozoans in the bradyzoite stage can induce cellular alterations leading to various types of host afflictions (Prandovszky et al., 2011). The presence of cerebral cysts is linked to the recruitment of the complement system protein C1q, which plays a pivotal role in eliminating protozoans (Xiao et al., 2016). However, its presence, particularly in excess, can disrupt neuronal synapses, potentially leading to neurological disorders (Xiao et al., 2016). *T. gondii* infection triggers the production of antibodies targeting N-methyl-D-aspartate (NMDA), a member of the ligand-gated ionotropic glutamate receptor subset, impeding proper synaptic propagation. Glutamate binding to NMDA receptors is vital for synaptic processes, especially learning (Lang et al., 2018; Zhu et al., 2018). This interaction is closely associated with neurological conditions such as Alzheimer’s disease and psychotic symptoms (Li et al., 2018), notably schizophrenia and bipolar disorder (Brooks et al., 2015).

*T. gondii* infection in the brain occurs mostly in neurons, leading to alterations in synapses, particularly glutamatergic and GABAergic synapses (Mouveaux et al., 2021). Importantly, GABA is an inhibitory neurotransmitter, and a deficiency in this neurotransmitter can result in various effects, one of which is the occurrence of seizures (Petroff et al., 1996). In addition to disrupting chemical synapses, infection triggers the activation of microglia, which, once activated, contribute to the loss of perisomatic inhibitory synapses via phagocytosis. This provides a potential mechanism for how infection by this pathogen may lead to seizures and psychiatric disorders (Carrillo et al., 2020).

The effects of infection also extend to other types of nerve cells beyond microglia, as shown by the reduction in the glutamate transporter GLT-1 in astrocytes. This leads to an increase in extracellular glutamate, which causes excitotoxicity (excessive activation of glutamate receptors, such as NMDA). This, in turn, generates a calcium overload in the cells, activating several intracellular signaling pathways that damage cellular structures, including reducing the number of dendritic spines. This is one of the mechanisms proposed to explain the dendritic reduction observed after *T. gondii* infection (David et al., 2016).

In addition to the more classic events described above, neurons infected with *T. gondii* cysts also exhibited alterations in cell–cell communication pathways, particularly through extracellular vesicles. Infected neurons showed reduced vesicle production, and their content was altered, containing proteins specific to *T. gondii*, such as GRA1, GRA2, GRA7, MAG1, and MAG2. Considering the intrinsic neuron–astrocyte communication, vesicles from infected neurons could also alter the gene expression of adjacent astrocytes (Tabaie et al., 2024).

These data highlight the significant impact of parasite infection on the brain parenchyma, with many pathways being redirected to support the success and benefit of the parasite. In fact, these chemical and physical changes have sparked discussions about whether they could be the cause of, or contribute to, the behavioral changes observed in rodents (Vyas et al., 2007) and psychiatric conditions, particularly schizophrenia. Recently, double impact theory has been proposed, suggesting early environmental risk. This affects the GABAergic, glutamatergic, or dopaminergic systems (as observed in *T. gondii* infection) and increases susceptibility to other risk factors later in life (theory reviewed by Guerrin et al., 2021).

## Endothelium and schizophrenia

The BBB is a complex structure, and as part of the neurovascular unit, cerebral endothelial cells form a protective barrier that helps maintain neuronal homeostasis. Dysfunction of the BBB might contribute to the pathogenesis of mental disorders such as depression, bipolar affective disorder, schizophrenia, dementia, intellectual disability and developmental disorders, including autism (World Health Organization, 2022a). These disorders affect approximately 1 in 4 people worldwide (World Health Organization, 2022b), with a combination of abnormal thoughts, perceptions, emotions and behaviors as clinical manifestations. Despite the heterogeneity of symptoms among them, it has been observed that cognitive dysfunction is a common pathway (MacQueen and Memedovich, 2017; Knight et al., 2018; Semkovska et al., 2019; Zhao et al., 2022).

Cognitive dysfunction is related to synaptic dysfunction; however, the adjacent factors that culminate in these events are still undetermined. Many researchers have suggested that inflammation and immune dysfunction can directly or indirectly contribute to neural networks (Deanna et al., 2018; Di Biase et al., 2021; Cui et al., 2023). This hypothesis of the brain–immune system axis was reinforced when an alteration in the permeability of the BBB was reported in mood disorders, autistic spectrum disorders and schizophrenia (Greene et al., 2020). Significant alterations, including basal lamina deformation, cytoplasmic vacuolization in endothelial cells (Uranova et al., 2010), and damage and loss of pericapillary oligodendrocytes in the prefrontal cortex (Vostrikov et al., 2008), has been observed in the postmortem brain of patients with schizophrenia.

In addition to structural dysfunction, the gold standard for assessing BBB permeability is the albumin ratio between CSF and blood. Since albumin levels are normally low in CSF, an increase indicates protein leakage from the blood into the CSF. Elevated CSF albumin levels have been observed in patients with schizophrenia (Bechter et al., 2010; Jeppesen et al., 2022; Campana et al., 2023). BBB permeability has been also observed through structural magnetic resonance imaging (Cheng et al., 2022; Moussiopoulou et al., 2023). This altered permeability facilitates the crossing of peripheral inflammatory factors, such as cytokines, across the CNS, which can trigger or ameliorate neural cell dysfunction. Among the cellular components, increased microglial activity, resulting from either external factors (such as peripheral cytokines) or internal factors (such as CNS infections), is a well-established risk factor for the development of mental illness (reviewed by Lurie, 2018).

In addition to the BBB, structural changes in the choroid plexus epithelium and the vascular endothelium have also been reported in pediatric patients (Zhou et al., 2020). Bitanihirwe et al (2022) reported an increased volume of the choroid plexus in patients with first-episode psychosis. Zeng et al. (2024) reported an enlargement in the choroid plexus of schizophrenia patients. These findings highlight significant morphological changes in patients with schizophrenia, with each component potentially contributing individually or collectively to the symptoms and progression of the disease.

Schizophrenia encompasses a wide range of symptoms, which can be categorized into three main types: positive symptoms (such as hallucinations and delusions), negative symptoms (including blunted affect, avolition, alogia and anhedonia) and cognitive symptoms (such as deficits in learning, memory and executive function) (American Psychiatric Association, 1994).

Schizophrenia patient-derived endothelial cells from induced pluripotent stem cells exhibit increased paracellular permeability *in vitro* (Stankovic et al., 2024). Among the paracellular mechanisms, tight junctions play a key role. Suppression of CLDN5 is associated with psychosis-like symptoms; deficits in learning, memory and sensorimotor control in mice (Greene et al., 2018); symptoms similar to those found in schizophrenia. These findings suggest that changes in the BBB could be linked to the patient’s symptoms. The expression of CLDN5, as measured by immunohistochemistry and quantitative PCR, is decreased in patients with schizophrenia and depression according to postmortem analysis (Greene et al., 2020; Lizano et al., 2022). In addition, high levels of IgA antibodies against CLDN5 have been detected in patients with schizophrenia (Maes et al., 2019), which is strongly suggestive of paracellular barrier disruption. It has not yet been elucidated whether the reduction in CLDN5 expression is the result of a single factor or a combination of factors. The contribution of matrix metalloproteinase-1 (MMP-1) to reducing CLDN5 expression has been reported (Rempe et al., 2018).

Endothelial cells from brain organoids derived from patients with schizophrenia exhibit alterations in angiogenic pathways and cell cycle regulation (Stankovic et al., 2024). Changes were observed in the ElF2, ID1, and mTOR pathways, with particular emphasis on the ID1 pathway, which activates HIF-1a and vascular endothelial growth factor-A (Stankovic et al., 2024).

Vascular endothelial growth factor (VEGF) levels have sparked significant scientific discussion; however, the findings remain conflicting. Pillai et al (2015) reported increased serum levels and VEGF expression in the parietal cortex, whereas Fulzele & Pillai (Fulzele and Pillai, 2009) reported reduced VEGF expression in the dorsolateral prefrontal cortex. Serum VEGF levels were lower in nonremitted first-episode psychosis patients than in remitted patients and healthy controls (Zhao et al., 2019). However, other studies have failed to detect significant differences in VEGF levels between patients with schizophrenia and healthy controls (Nguyen et al., 2018; Pu et al., 2020). An explanation for these differences is directly related to patient condition, since VEGF levels change with the use of antipsychotics. Medication-free patients have decreased serum VEGF levels, which tend to increase with treatment (Lee et al., 2015; Xiao et al., 2018; Xiao et al., 2019). VEGF is produced by different cells, such as astrocytes, neurons, endothelial cells and microglia; therefore, understanding its expression at the brain level would be interesting since it plays a role in modulating the BBB (Proescholdt et al., 1999). VEGF controls the expression of tight junction proteins such as CLND5 and occludin, downregulating their expression, leading to BBB disruption (Argaw et al., 2009) and facilitating the infiltration of peripheral cells into the CNS (Cai et al., 2020a).

The entry of peripheral cells is facilitated by the expression of adhesion molecules, such as selectins, ICAM-1 and VCAM-1. The expression of these adhesive molecules is altered in patients with schizophrenia, depending on the stage of the disease and type of treatment (Cai et al., 2020a). The serum level of soluble ICAM-1 (sICAM-1) is lower in patients before the initiation of antipsychotic therapy than in controls (Schwarz et al., 2000; Krönig et al., 2005; Kavzoglu and Hariri, 2013; Radu et al., 2020). Although Schwarz et al (2000) did not observe a difference in sICAM-1 levels after therapy, several studies have reported an increase in sICAM-1 levels following treatment (Meyer et al., 2009; Kavzoglu and Hariri, 2013; Cai et al., 2020b; Radu et al., 2020). Elevated sICAM-1 levels have been observed in chronic patients receiving continuous medication (Nguyen et al., 2018; Cai et al., 2020a; Sheikh et al., 2023).

The data regarding soluble VCAM (sVCAM) levels in patients with schizophrenia are more conflicting. Kavzoglu & Hariri (Kavzoglu and Hariri, 2013) did not find a difference in plasma sVCAM levels between patients with first-episode psychosis and controls. They reported that, after drug therapy, the levels of this molecule remained at baseline (Kavzoglu and Hariri, 2013). Moreover, Radu et al. (2020) initially detected increased sVCAM levels in patients with schizophrenia, which began to decrease after therapeutic intervention. In contrast, Meyer et al. (2009) did not observe any differences in sVCAM levels between patients receiving antipsychotic therapy and controls.

Stefanović et al. (2016) explored the differences in these adhesion molecules during the different stages of the disease, following the initial phase (period until 3 years after first-episode psychosis) and final phase (minimum of 10 years after the diagnosis of schizophrenia). To minimize interfering variables, only patients who were not on major psychotropic drugs for at least four weeks prior to hospitalization were included. During the initial phase, they detected normal levels of sICAM1 and reduced levels of sVCAM, whereas in the late phase, they reported that the levels of sICAM increased and those of sVCAM decreased (Stefanović et al., 2016). sICAM-1 originates from proteolytic cleavage of membrane ICAM-1 or alternative mRNA ICAM-1 splicing and has a wide tissue distribution (King et al., 1995). Thus, the increase in sICAM-1 provides a general picture of inflammation, whereas the increase in sVCAM is related to endothelial dysfunction (Radu et al., 2020).

Alterations in soluble P-selectin (sP-selectin), a molecule that slows leukocytes on the endothelial surface before they bind tightly to cellular adhesion molecules, was also observed in patients with schizophrenia (Pinjari et al., 2022). Patients with schizophrenia had a positive correlation between sP-selectin levels and the astrocytic marker S100B (Pinjari et al., 2022). S100B is a calcium-binding peptide produced primarily by astrocytes. As its concentration is practically undetectable in healthy individuals, an increase in its level is associated with pathologies that disturb the BBB. Indeed, some studies have detected elevated levels of this marker in the blood, CSF and brain of patients with schizophrenia (Wiesmann et al., 1999; Lara et al., 2001; Schroeter et al., 2003; Kealy et al., 2020).

There is also a positive correlation between sP-selectin and interleukin 6 (IL-6) in patients with schizophrenia (Pinjari et al., 2022). Pillai et al. (2015) reported a positive correlation between increased serum levels of VEGF and IL-6 in patients with schizophrenia. Taken together, these data support the hypothesis that inflammation may be an important contributor to the etiology of schizophrenia (Fan et al., 2007; Potvin et al., 2008; Drexhage et al., 2010).

The upregulation of mRNAs and proteins related to the inflammatory response is found in patients with schizophrenia (Zhu et al., 2022). A subgroup of patients with schizophrenia shows an exaggerated increase in the mRNA levels of inflammatory cytokines, such as IL-1β, IL-6, IL-8 and SERPIN3, in the frontal cortex; these patients are designated high-profile inflammatory patients (Fillman et al., 2013; Zhang et al., 2016). The peripheral cytokine profile is used to classify patients with schizophrenia into high or low inflammatory biotypes (Fillman et al., 2016) (Cai et al., 2020a). Patients with a high inflammatory profile present important neuronal alterations, such as cognitive deficits, a reduction in brain volume (Fillman et al., 2016), the presence of hypertrophic astrocytes (Catts et al., 2014), and a reduction in cortical gray matter volume (Zhang et al., 2016).

The links among inflammation, cognitive impairments, and brain changes suggest that targeting inflammatory pathways is a promising treatment for schizophrenia, particularly in high-inflammatory patients with severe neurological deficits. Inflammation may increase BBB permeability and neuronal function, leading to dysfunction of the neurovascular unit and contributing to disease progression. Additionally, the role of infectious agents in immune dysfunction warrants further study, offering insights into the complex causes of schizophrenia and potential targeted therapies.

## Relationship between schizophrenia, toxoplasmosis and endothelium

Chronic infection by *T. gondii* results in decreased synaptic density, neural apoptosis, a reduction in dendritic arbor and spine density and abnormalities in neurotransmitters (such as increased dopamine) due to excessive secretion of cytokines from microglia and astrocytes (Parlog et al., 2014; Wang et al., 2019). This is accompanied by increased extracellular glutamate and reduced glutamate transporter activity in glial cells (David et al., 2016). Other synaptosomal proteins, such as EAAT2, Shank3, AMPA and NMDA receptors, are also dysregulated (Lang et al., 2018). These events help in understanding the behavioral changes in the host caused by *T. gondii* (Barrios et al., 2021). Some of those findings are similar in patients with schizophrenia. Decreased synapse density (Van Berlekom et al., 2020), increased dopamine release (McCutcheon et al., 2019), NMDA hypofunction (Weickert et al., 2013) and apoptosis due to microglial activation (Parellada and Gassó, 2021) are some of the biological findings in schizophrenia. An example of the infection process and the access of *T. gondii* to neurons and some of its consequences are shown in **Figure 1**.

Studies carried out around the world reported a higher seroprevalence of anti-*T. gondii* antibodies in populations with schizophrenia than in healthy populations (Cetinkaya et al., 2007; Dogruman-Al et al., 2009; Yuksel et al., 2010; Alipour et al., 2011; Alvarado-Esquivel et al., 2011; Fond et al., 2018; Burgdorf et al., 2019; Morais et al., 2019; Stepanova et al., 2019; Kezai et al., 2020; Ekici et al., 2021; Galván-Ramírez-M-de-la et al., 2021; Grada et al., 2022; Omer, 2022; Lili et al., 2024). The relationship between this pathogen and schizophrenia has been explored, and patients with schizophrenia exhibit a more inflammatory state that can lead to altered permeability of the BBB, and this event can be exacerbated by *T. gondii* infection (Zhu et al., 2022). In addition to acute infection, the persistence of parasitic cysts in the brain promotes chronic inflammation, which in turn leads to changes in the BBB, facilitating the entry of cytokines from the bloodstream into brain tissue (Barrios et al., 2021). *T. gondii* seropositivity is linked to increased levels of neuron-specific enolase and IL-18 (Andreou et al., 2024). IL-18 is particularly noteworthy, as it can positively regulate the production of INF-γ (Berg et al., 2002). In turn, IFN- γ not only is involved in the immune response against the parasite but also promotes an increase in adhesion molecules, such as ICAM-1 (Jahnke and Johnson, 1995), which is also altered in schizophrenia. In this context, Pinjari et al. (2022) reported that sP-selectin is positively correlated with interleukin 6 (IL-6). A study evaluating the function of BMECs revealed an increase in CCL2 in a group exhibiting a BBB deficit, which was observed in a subgroup of patients with SZ or bipolar disorder (BD) (Lizano et al., 2023). CCL2, along with P-selectin, IL-6, TNF-α, CXCL9, MMP-8 and MMP-13, also appears to be deregulated in *T. gondii* infection (Lachenmaier et al., 2011; Figueiredo et al., 2022).

Although overlapping pathways can be found in these pathologies, it is still undetermined which events occur first. In this context, two scenarios can be defined. First, it could be hypothesized that patients with schizophrenia are more susceptible to infection because they have a greater inflammatory state, with the upregulation of adhesion molecules (ICAM-1, sP-selectin) and chemokines (such as CCL2). The importance of ICAM-1 stands out here, as it is the main molecule responsible for the transmigration of immune cells to the brain parenchyma (Cai et al., 2020b). In addition, the neutrophil–lymphocyte ratio in the blood of patients with schizophrenia is elevated (Semiz et al., 2014; Šagud et al., 2023; Liang et al., 2024). Therefore, when these individuals are infected by *T. gondii*, not only are greater quantities of cells available for the parasite to infect but also the chances of parasitized cells (in the Trojan horse strategy: see **Figure 1**) entering the brain are greater. Once infection is established in the nervous system, it can exacerbate the neuroinflammation observed in patients with schizophrenia (Doorduin et al., 2009).

The second scenario suggests that the infection occurred prior to the outcome of schizophrenia (Niebuhr et al., 2008; Sutterland et al., 2015). In this context, parasite infection leads to the production of antibodies, and since peptide overlap exists between *T. gondii* and the NMDA receptor subunits, it could lead to a cross-reaction between anti-*T. gondii* antibodies and NMDA receptor subunits (Lucchese, 2017), resulting in NDMA receptor dysfunction. In fact, one of the arms that seeks to explain the etiology of schizophrenia is dysfunction of this same receptor (Northoff et al., 2005).

In addition, after activation by IFN-γ, astrocytes and microglia become the main effector cells in defense against the parasite via the secretion of IL-1, IL-6, GM-CSF or IL-10 and TNF-α, respectively (Maenz et al., 2014). TNF-α and IL-6 are associated with an increase in MMP-1 (Du et al., 2016). This can activate other MMPs, such as MMP-2 and MMP-9, which can reduce the expression of tight junction proteins associated with BBB disruption (Rempe et al., 2018). In addition, individuals with schizophrenia have high levels of MMP-9 (Domenici et al., 2010; Yamamori et al., 2013; Devanarayanan et al., 2016; Ali et al., 2017).

**Figure 2:**
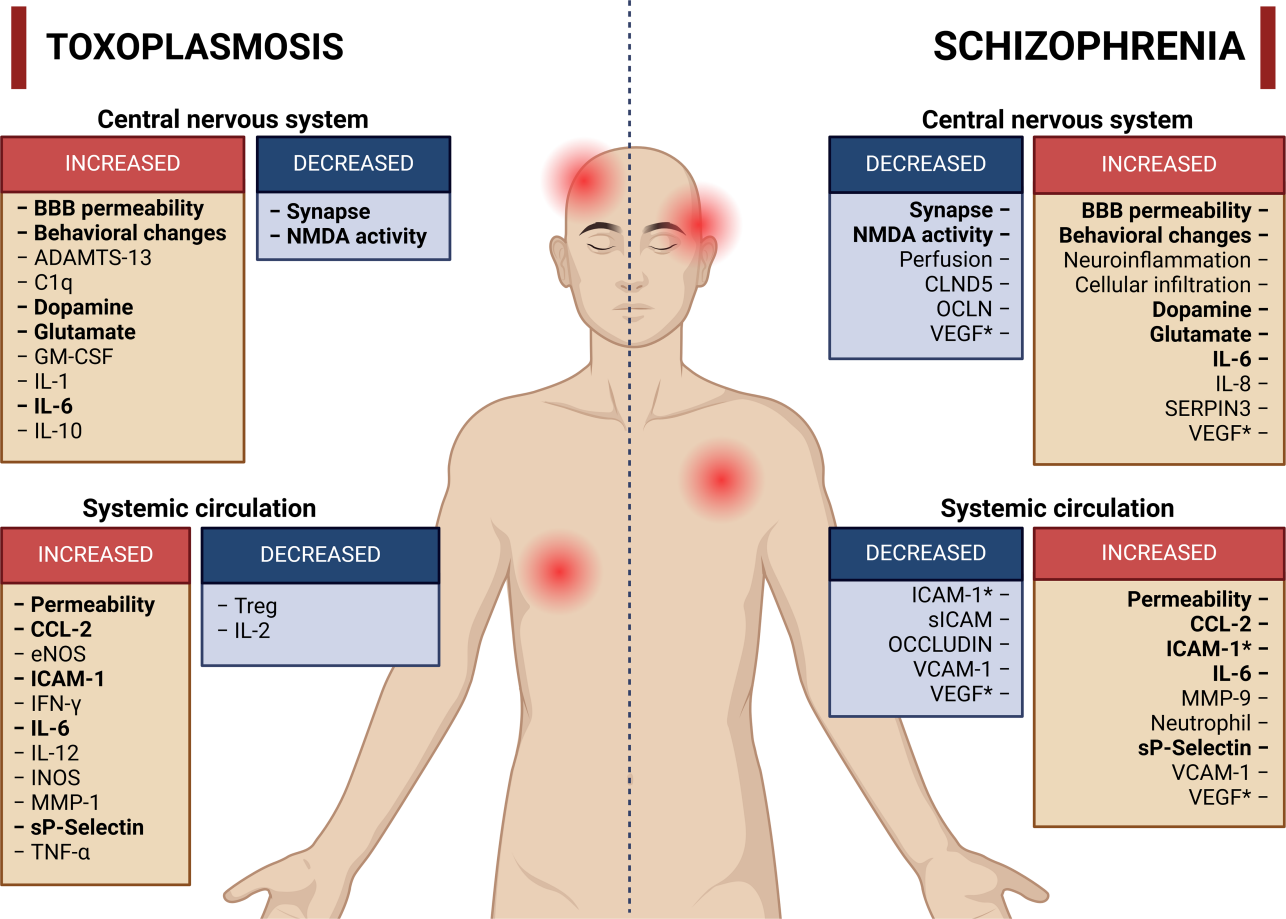
Main activities or molecules altered in toxoplasmosis and schizophrenia. The red boxes represent activities or molecules with increased concentration or activity, whereas the blue boxes represent molecules or activities with reduced concentration or activity. The terms highlighted in bold represent activities or molecules with similar increases or reductions in both toxoplasmosis and schizophrenia. References have been omitted from this figure to facilitate visualization. *Increase or decrease in the number of molecules according to different studies. Created in BioRender. Albrecht, L. (2024). https://BioRender.com/g51d357.

Therefore, inflammatory processes, as discussed in the previous paragraph, may help explain the existence of a subgroup of patients with schizophrenia who have altered BBB permeability (Najjar et al., 2017; Cai et al., 2020a; Hoang et al., 2022), as well as subtypes of patients with a greater inflammatory state (Bechter et al., 2010; Kunz et al., 2011; Fillman et al., 2013; Fillman et al., 2016). Chronic infection contributes to the establishment of constant neuroinflammation that can impair CNS homeostasis (de Medeiros Brito et al., 2020; Meurer Y da et al., 2020; Brito Rm de et al., 2023). A brief summary of the changes identified in individuals infected with *T. gondii* compared with individuals with schizophrenia is shown in **Figure 2**.

On the basis of these findings, the potential for *T. gondii* infection to trigger neuroinflammation and disrupt NMDA receptor function raises the possibility that infection could contribute to the development of schizophrenia or exacerbate preexisting conditions. Furthermore, the overlap between *T. gondii* antigens and NMDA receptor subunits, leading to a cross-reaction of antibodies, could provide insight into the mechanisms underlying NMDA receptor dysfunction in schizophrenia. These findings highlight the need for further research into the interaction between *T. gondii* and schizophrenia, particularly in exploring whether the infection acts as a trigger or an exacerbating factor. Such research could suggest therapeutic strategies aimed at modulating neuroinflammation and restoring BBB integrity in affected individuals.

## Perspectives

This review aimed to investigate the interaction between toxoplasmosis and schizophrenia, exploring the factors contributing to the variability in clinical outcomes observed in patients with schizophrenia. By highlighting these discrepancies, we sought to guide the design of future studies by considering variables such as medication, patient age, and the presence or absence of infectious diseases. Given the multifaceted nature of psychiatric disorders, our goal was to contribute to the understanding of the complex puzzle by examining the associations among *T. gondii* infection, endothelial interactions, and the immune system.
